# Complete Reference Genome Sequence of the Extensively Drug-Resistant Strain Neisseria gonorrhoeae AT159, with Ceftriaxone Resistance and High-Level Azithromycin Resistance, Using Nanopore Q20+ Chemistry and Illumina Sequencing

**DOI:** 10.1128/mra.00744-22

**Published:** 2022-08-25

**Authors:** Daniel Golparian, Sonja Pleininger, Susanne Jacobsson, Alexander Indra, Magnus Unemo

**Affiliations:** a World Health Organization Collaborating Centre for Gonorrhoea and Other Sexually Transmitted Infections, Department of Laboratory Medicine, Microbiology, Faculty of Medicine and Health, Örebro University, Örebro, Sweden; b Austrian Agency for Health and Food Safety, Vienna, Austria; c Institute for Global Health, University College London, London, United Kingdom; University of Maryland School of Medicine

## Abstract

Extensively drug-resistant Neisseria gonorrhoeae (XDR-NG) strains with resistance to the last remaining first-line treatments (ceftriaxone monotherapy or combined with azithromycin) represent the emerging threat of untreatable gonorrhea. We present the complete reference genome sequence of the XDR-NG strain AT159, with ceftriaxone and high-level azithromycin resistance, from Austria.

## ANNOUNCEMENT

Neisseria gonorrhoeae (NG) has developed resistance to all antimicrobials recommended for gonorrhea treatment. Ceftriaxone 0.25 to 1 g (monotherapy) or ceftriaxone 0.25 to 1 g plus azithromycin 1 to 2 g (dual therapy) are the last remaining first-line treatments worldwide (1, 2). Recently, a second extensively drug-resistant NG (XDR-NG) strain (AT159) with ceftriaxone resistance plus high-level azithromycin resistance caused a possible gonorrhea treatment failure in Austria (3).

AT159 was diagnostically cultured on modified Thayer-Martin agar medium (36  ±  1°C, 5% CO_2_, 24 h) from the urethra of an Austrian male (infected in Cambodia) in April 2022 (3). Prior to extraction of genomic DNA using the Nanobind CBB Big DNA kit (Circulomics), AT159 was cultured on chocolate agar medium (36  ±  1°C, 5% CO_2_, 24 h). Nanopore (Oxford Nanopore Technologies) sequencing was performed without DNA fragmentation, and short fragments (<3 kb) were excluded using long fragment buffer (Nanopore). The Nanopore sequencing library was prepared using the duplex Nanopore Q20+ chemistry (SQK-LSK112) and sequenced on an Mk1C instrument for 20 h using a R10.4 (FLO-MIN112) flow cell. Bases were called using ont_guppy_duplex_pipeline v1.0.0 with the super high accuracy (SUP) option, generating 106,806 (21,899 duplex) reads with a Phred quality score of >Q30 and an *N*_50_ read length of 37,130 bp. Adapters were trimmed using Porechop v0.2.4 (https://github.com/rrwick/Porechop). Illumina DNA prep and the NextSeq 550 (Illumina) platform were used for short-read sequencing, with an average depth of 240×. The DNA was sheared using enzymatic fragmentation (average fragment size, 452 bp) using the provided magnetic beads, according to Illumina protocols, and sequenced without any size selection. All reads were quality controlled using CLC Genomics Workbench v22.0.1 (4), and low-quality (<Q30) bases were removed. Kraken v1.1.1 (http://ccb.jhu.edu/software/kraken/) was used for species confirmation. Hybrid assembly using the Nanopore and Illumina reads was performed using Unicycler v0.4.7 (5), which was also used to rotate the single contig to start with DnaA (UniProtKB accession number Q9JXS7) and the circularized plasmids to start with MobC and TrbF for the cryptic plasmid and conjugative plasmid, respectively. Default parameters were used for all software unless otherwise specified.

The complete AT159 chromosome was 2,232,771 bp (G+C content, 52.4%). The Prokaryotic Genome Annotation Pipeline (PGAP) v6.1 (6) was used to annotate 2,335 coding sequences, 55 tRNAs, and 12 rRNAs. AT159 also harbored cryptic (4,197 bp) and *tetM*-carrying conjugative plasmids (41,997 bp; Dutch plasmid type). AT159 was resistant to ceftriaxone (MIC, 0.25 mg/L), cefotaxime (MIC, 0.5 mg/L), cefixime (MIC, 1 mg/L), azithromycin (MIC, >256 mg/L), ciprofloxacin, and tetracycline (3). The genome contained many resistance determinants causing this XDR-NG profile, e.g., mosaic *penA*-60.001, 23S rRNA A2059G, *gyrA* S91F/D95G, and *tetM* (1). Genomic core analysis (2,062 genes), using SeqSphere v8.3.1 (7), of the majority of publicly available gonococcal genomes in ENA (*n* = 31,945) revealed that AT159 is relatively closely related (1,919 single nucleotide polymorphisms [SNPs]) to the only previously described XDR-NG strain with ceftriaxone and high-level azithromycin resistance (WHO Q) (8). However, AT159 was distant from the internationally spreading ceftriaxone-resistant clade FC428 (9) (4,435 SNPs) and the first described ceftriaxone-resistant XDR-NG strains (WHO X and Y [10]).

Isolate AT159 was cultured using routine diagnostics (standard care). No ethical approval was required to examine and publish the isolate without any personally identifying data from the patient. Nevertheless, the patient gave consent to publication (3).

### Data availability.

The complete sequences were deposited at GenBank under accession numbers CP097846.1 (chromosome) and CP097847.1 and CP097848.1 (plasmids). The raw reads are available through the NCBI Sequence Read Archive under accession numbers SRX15948642 (Illumina) and SRX15948643 (Nanopore). The project summary can be found under BioProject accession number PRJNA839941.
